# Perceiving Numbers Affects the Internal Random Movements Generator

**DOI:** 10.1100/2012/347068

**Published:** 2012-04-26

**Authors:** Carmelo Mario Vicario

**Affiliations:** Cognitive Neuroscience Sector, SISSA, Via Bonomea 265, 34136 Trieste, Italy

## Abstract

According to the evidence of direct relationships among space, numbers, and finger representations, a random movement generation (RMG) task was employed in order to investigate whether numerical exposure can influence the finger selection of healthy humans. To this purpose a group of participants were asked to generate random finger movements during the exposure to several numerical cues. Although participants were explicitly asked to move finger as random as possible, results showed that left-hand fingers were moved more frequently than right-hand fingers when low numerical cues (from 1 to 3) were presented, and, *vice versa*, right-hand fingers were moved more frequently than left-hand fingers when high numerical cues (ranged from 7 to 9) were presented. The current result suggests that spontaneous actions can be affected by abstract information, providing an evidence that numerical concepts can influence low-level, non-goal-directed behaviours.

## 1. Introduction

Free will, probably the highest expression of the human being, has an immediate impact in programming and executing everyday life behaviours. Freedom has been defined the ability to consciously decide how to act. This implies the necessity to be conscious of one's own decision making, to be free [1]. *Libertarians *suggest that our conscious intentions cause our actions [2], and this view admits that the person, her- or himself, is an essential element in the determination of free actions. On the other hand, *compatibilists* claim that freedom and natural causality might coexist [2], while *no freedom *theorists intend the subjective experience of freedom as no more than an illusion, since our actions are initiated by unconscious mental processes long before we become aware of our intention to act [2, 3]. Therefore, according to the *no freedom *view, volition must be intended as awakened to external and ungovernable forces, especially when people are unaware of them. Cognitive neuroscientists have already shown interest in this issue by studying the activation of parietal neurons in non human primates asked to choose among several possible alternative options. It was shown that neurons of this brain area, which are also known to be involved in planning eye movements ([4], see [5]), seem to fire before one choice is made to generate a movement [6]. Inputs from this area, in turn, would modulate the activity of frontal regions and, ultimately, help to decide which movements will be made [4].

Parietal activation was also widely reported in subjects asked to perform a simple number processing task [7], as well as during tasks requiring a spatial processing (see [8], for a complete review). A subregion of the parietal cortex, the intraparietal sulcus (IPS), seems to be crucially activated when spatial updating and number processing are involved. Accordingly [9], suggested that a nonverbal representation of numerical quantity, perhaps analogous to a spatial map or “mental number line,” is present in the IPS of both hemispheres. The mental number line reflects a metaphor positing that low numbers are associated with left-side space and higher numbers with right-side space [10]. This model accounts for a faster left-hand response when numbers are relatively small and, *vice versa*, a faster right-hand response when numbers are relatively large. In consideration of the above discussed overlaps between areas activated when a decision is going to be made, quantity representation and spatial encoding, the current study was designed in order to address the question of whether task-irrelevant cues such as visual digit are able to affects the spontaneous motor behaviour. In a previous study, Daar and Pratt [11] manipulated numerical magnitude and manual response in order to investigate the presence of a compatibility effect in the response selection. Their results showed that low digits biased the voluntary selection of typing with their left hand, while high digits biased the voluntary selection of typing with their right hand.

In the current research I expanded this paradigm by using a random movement generation (RMG) task, a behavioral paradigm which is used to test a wide amount of cognitive functions such as supervisory control for trial-by-trial decision-making, inhibition of habitual responses, switching of response strategies, and shift of attentional focus [12]. This investigative approach was supported by the results coming from a recent research which has successfully employed the random number generation (RNG) task for exploring the properties of numerical spaces [13]. These authors were able to show a decisional bias in the numerical size selection by manipulating sensorimotor coordinates: specifically, while facing left, subjects produced relatively small numbers, whereas while facing right they tended to produce relatively larger numbers [13]. By using an RMG, the purpose of the current research was to investigate whether numerical size affects the random generation of finger movement sequences. In contrast with the randomness of performing the task, I predicted that the frequency with which participants would generate left- or right-hand finger movements would be modulated by the magnitude of the number displayed on the screen. In particular, I expected that left-hand finger movements will be selected more frequently than right-hand finger movements when low numbers (from 1 to 3) are displayed; *vice versa*, right-hand finger movements will be selected more frequently than left-hand finger movements when high numbers (ranged from 7 to 9) are presented. No difference is expected for middle numbers (ranged from 4 to 6).

## 2. Materials and Methods

### 2.1. Participants

Seventeen right-handed participants (5 men, 12 women, mean age: 24.05 ± 1.95 years) with normal or corrected vision participated in the research after providing written informed consent. All participants were Italian native speakers. They received a reward payment of 7 Euros.

### 2.2. Procedure and Instruments

**Figure 1:**
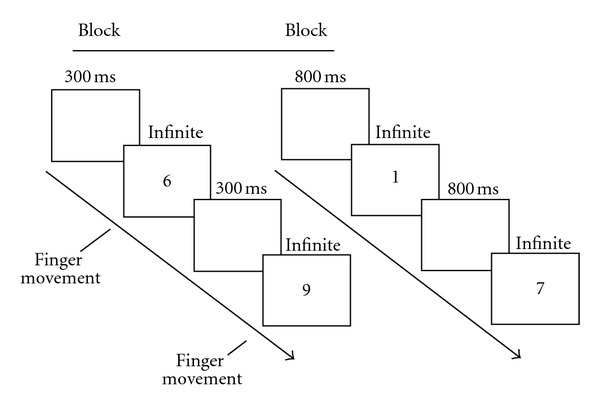
Example of typical trial sequence.

Participants were positioned 50 centimetres from an Olidata computer monitor configured at a refresh rate of 100 Hz. Visual stimuli were composed of nine numerical cues (from 1 to 9, size 0.8° × 0.1°). Numbers were casually presented in two separate and consecutive blocks (counterbalanced design) according to two precise Inter Stimulus Intervals (ISIs = 300 ms or 800 ms). These intervals marked the temporal peace for the finger movements. Participants were explicitly asked to synchronize their responses with the numerical cue displacement. This modality to present numerical cues was programmed in order to simulate a visual metronome. Participants were asked to respond to numerical cues by pressing one among 8 keys of the keyboard (A, S, D, F, H, J, K, L) with one of their eight fingers (the index, the middle, the ring, and the pinkie of both left and right hands). The “go” signal to move a finger was represented by the numerical cue itself. The numerical cue disappeared once the participant pressed the selected key. Each block consisted of a total of 90 trials (10 per numerical cue) displayed on the centre of the computer screen. The dependent variable was the frequency with which a finger movement selection was made following the presentation of low (from 1 to 3), middle (from 4 to 6), and high numbers (from 7 to 9). See Figure 1 for further details.

### 2.3. Data analysis

The dependent variable was the frequency of finger movements made with both the left and the right hands during the displaying of all the digits. The amounts of finger movements generated were analyzed by using a repeated measures ANOVA with 8 finger movements (4 left and 4 right) 3 numerical sizes (low, medium, high) × 2 inter stimulus intervals (ISIs, 300 ms and 800 ms) as factors were performed to assess the interaction between the magnitude of numbers and finger movement. Post hoc comparisons were performed using unpaired *t*-test. The percentages of movement frequency were fit with a linear regression (*y* = *ax* + *y*_0_), and the slope and intercept values obtained for both groups were compared. Data analysis was performed using the STATISTICA software, version 8.0, StatSoft, Inc., Tulsa, USA.

## 3. Results

**Figure 2:**
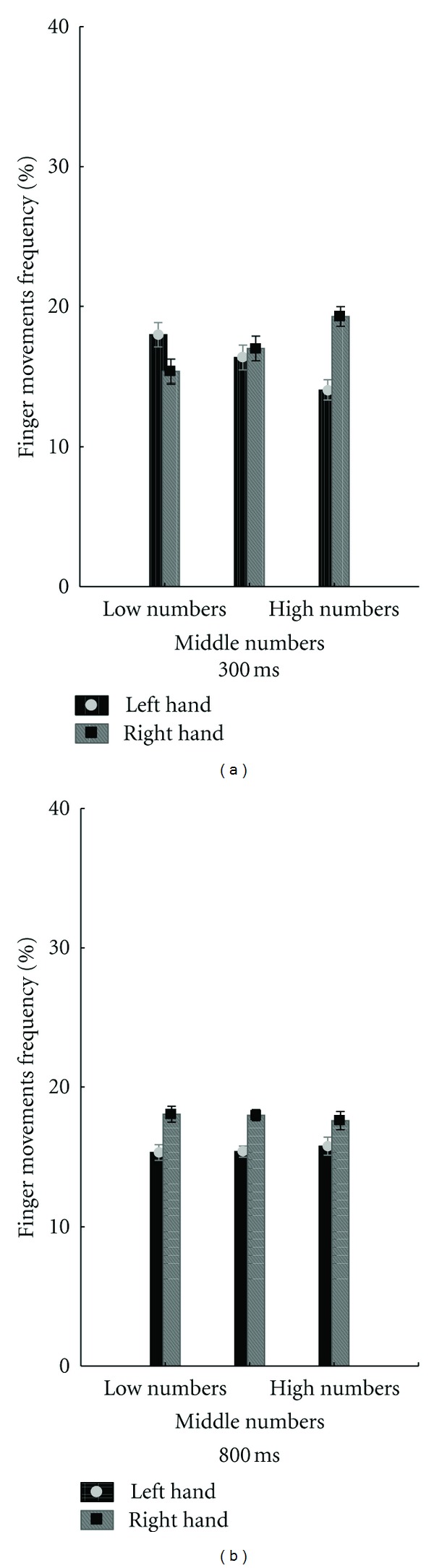
Influence of numerical magnitude on random movement generation. The graph plots the percentage of finger movements on the exposure to high (ranging from 7 to 9), middle (ranging from 4 to 6), and low numbers (ranging from 1 to 3). The interstimulus intervals were set up at 300 and 800 milliseconds (ms). Vertical bars indicate standard error. *indicates significant differences, *P* level = 0.05.

A significant main effect of finger movement factor was observed (*F*(1,16) = 21.2, *P* < 0.001) with participants producing more finger movements with their right hand (*M* = 15.78 ± 0.170) with respect to their left hand (*M* = 14.21 ± 0.170). The Finger Movement ∗ Numerical Size ∗ ISI interaction was also significant (*F*(2,32) = 3.76, *P* = 0.034). Post hoc comparisons revealed that, when the ISI of numerical cues were at 300 ms, right finger movements (*M* = 17.35 ± 0.629) were significantly more frequent than left finger movements (*M* = 12.64 ± 0.629) during the exposure to high numbers (*t*(1,16) = 5.28, *P* = 0.001). On the other hand, left finger movements (*M* = 16.17 ± 0.782)  were more frequent than right finger movements (*M* = 13.82 ± 0.355) during exposure to low numbers (*t*(1,16) = − 2.12, *P* = 0.020). No differences were reported by comparing right finger movements (*M* = 15.29 ± 0.798) with left finger movements (*M* = 14.70 ± 0.798) (*t*(1,16) = 0.52, *P* = 0.303) during exposure to middle numbers (Figure 1). When the ISI of numerical cues was at 800 ms, there was a significant difference in the movements frequency between right (*M* = 15.82 ± 0.583) and left (*M* = 14.17 ± 0.798) fingers (*t*(1,16) = 1.99, *P* = 0.026) during the exposure to high numbers. Likewise a significant difference was reported by comparing right finger movements (*M* = 16.17 ± 0.355) with left finger movements (*M* = 13.82 ± 0.355) during exposure to low numbers (*t*(1,16) = 4.67, *P* < 0.001). Furthermore, a significant difference in the movement frequency was observed by comparing right finger movements (*M* = 16.23 ± 0.511) with left finger movements (*M* = 13.76 ± 0.511) during the exposure to middle numbers (*t*(1,16) = 3.41, *P* = 0.001) (Figure 2).

All the other main effects ISI [*F*(1,16) = 0, *P* = n.a.], numerical size [*F*(2,32) = 0; *P* = n.a.], or interaction terms, ISI ∗ finger movements [*F*(1,16) = 1.65; *P* = 0.216] ISI * numerical size [*F*(2,32) = 0; *P* > 0.05] and finger movement ∗ numerical size [*F*(2,32) = 2.91; *P* = 0.068] were not significant.

In order to explore whether the numerical size predicts the movement frequency for each finger, eight separated regression analysis were performed on the responses block in which the ISI of numerical cues was at 300 ms. Pinkie, ring finger, middle finger, index finger of the left hand corresponded to the “A,” “S,” “D,” “F” letters, respectively (A: *y* = 21.3333−0.6667 ∗ *x*, *r* = −0.4637, *P* = 0.2086; S: *y*  = 28.3889−0.8333 ∗ *x*; *r* = −0.4103, *P* = 0.2728; D: *y* = 27.5833−0.7833 ∗ *x*;  *r* = −0.6870, *P* = 0.0409; F: *y*  = 21.5833 −0.3833 ∗ *x*, *r* = −0.3452, *P* = 0.3629).

**Figure 3:**
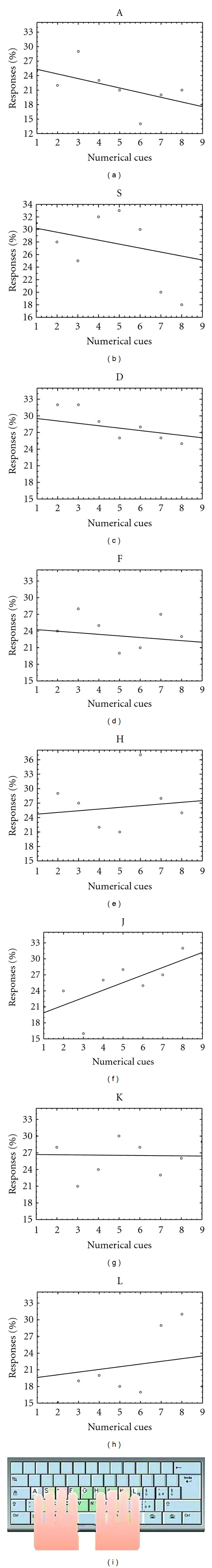
The figure plots the percentage average of finger movements for the eight keys of the keyboard during the exposure to the nine numerical cues at 300 msec of ISI. The ordinate represents the proportion of responses generated with a finger; the abscissa represents the displayed numerical cue.

Pinkie, ring finger, middle finger, index finger of the right hand corresponded to the “H,” “J,” “K,” “L” letters, respectively (H: *y* = 19.2778 + 0.6333 ∗ *x*; *r* = 0.3634, *P* = 0.3364; J: *y* = 14.7222 + 1.3 ∗ *x*; *r* = 0.7515, *P* = 0.0196; K: *y* = 20.9722 + 0.25 ∗ *x*; *r* = 0.2171, *P* = 0.5747; L: *y* = 16.1389 + 0.4833 ∗ *x*; *r* = 0.2489, *P* = 0.5184). Results are summarized in Figure 3.

## 4. Discussion

Discussing free will in the light of the causality—the idea that motor behaviour is caused by prior events—I have addressed the question of whether the exposure to numbers of different sizes has immediate sensorimotor consequences on action selection(e.g., [14]). Previous studies have extensively shown that, in both forced [10] and free-response [11] paradigms, perceiving numbers affects motor performance. On the other hand, the RMG task used in the current research embraces both of these features (freedom and constraint) since it requires forced responses in a free context. In fact participants had to respond as randomly as possible, but, at the same time, they were forced to synchronize their responses by using a defined temporal pace. According to previous evidences [11], the current results have shown that the higher the numerical cue the higher the probability of using right-hand fingers and, vice versa, the lower the numerical cue the higher the probability of using left-hand fingers. No left-right difference in the finger movement frequency was reported for the exposure to middle numbers. However, the motor ∗ numerical interaction was selectively found when the ISI of the numerical cues was fixed at 300 ms, while no number ∗ finger movements interaction was reported when setting the ISI at 800 ms. In this last case, in fact, right-hand fingers were moved more frequently than left-hand fingers, independently of the numerical cue displayed on the computer screen. The regression analysis has shown a negative trend between left finger movement's frequency and numerical size and, vice versa, a positive trend between right finger movement's frequency and numerical size. However, although the negative trend for all left finger movements and the positive trend for all right finger movements, statistical analysis has shown significant results only for movements generated with fingers corresponding to the letters “D” and “J” of the keyboard. Taken together these results suggest the following conclusions.

- The numbers/finger movement interaction takes place in a very early period. In fact, the current results have shown a significant interaction when the ISI of numerical cues was set up at 300 ms while no interaction was reported at 800 ms.
- Middle numbers balance the finger movements frequency across hands when the ISI of numerical cues is set up at 300 ms. In fact, no finger movements difference across hands was reported during the exposure to numerical cues ranging from 4 to 6. This result is in contrast with that reported when the ISI of the numerical cues was set up at 800 ms in which it was found a greater tendency of participants to move their right fingers, regardless of the size conveyed through the numerical cue.
- The size of numerical cues predicts the frequency of finger movements direction since the regression analysis has shown a negative trend between the frequency of left finger movements and the numerical size and, vice versa, a positive trend between the frequency of right finger movements and the numerical size.

In a seminal study, Baddeley and colleagues [15] have shown a reduction of randomness in the free movement generation task when participants were asked to execute a secondary task requiring a change of attentional set or switching. Likewise, the modulation of attentional mechanisms in space can explain how numerical information influences the participants' spontaneity in generating random finger movements. In fact, the mere sight of a number would induce a spatial attentional bias, which depends on its magnitude, with low numbers shifting attention to the left and high numbers shifting attention to the right space [16].

According to this suggestion, the spatial attention modulation toward the left and the right space generated by the numerical exposure would be responsible of the current phenomenon. On the other hand, the lack of numbers/finger movements interaction when the ISI was set up at 800 ms and the number finger interaction when the ISI was set up at 300 ms indicate a possible violation for the spatial attention hypothesis. A previous study, in fact, has clearly reported that numbers can affect spatial attention when the stimulus onset asynchrony was set up between 500 ms and 750 ms [16]. This fact indicates that other factors, beyond the spatial attention modulation, might underlie the number ∗ finger interaction reported in the current study. A possible suggestion is that the motor ∗ numerical interaction that was found when the ISI was set up at 300 ms originates from an early competition for cognitive resources between decisional processes engaged when planning a left-to-right finger movements selection (as required by the task procedure) and those engaged for a left-to-right spatial representation of numbers, as proposed by the mental number line model [10]. This interpretation seems to have an anatomical rational as demonstrated in a recent imaging study showing that numerical processing activates a frontoparietal cortical network that partly overlaps regions associated with the control of finger movements [17].

Previous evidences have documented effects of the numerical exposure on the generation of several types of movement [18, 20]. The current result expands these findings by showing that task-irrelevant numerical exposure predictably biases the spontaneous generation of spatially encoded movements, against the participants' efforts to be casual. The fact that numerical exposure affects the motor behaviour by modulating decisional processes is consistent with evidences suggesting a role of the parietal cortex in statistical decision-making processes [21]. Within the parietal cortex, the lateral intraparietal (LIP) area appears as the probable candidate possessing response properties that are related to both numerical and spatial processing [22].

The current finding represents also an interesting contribute to address the current debate between embodied and disembodied theories of cognition since it provides the opposite side of the interplay between cognition and sensorimotor systems (e.g., [23, 24]): while the embodied cognition view proposes that body states and situated action underlie cognition [25], disembodied theories of cognition [26] reject this view embracing the hypothesis that the mind is the result of a computation on amodal symbols in a modular system. In the light of this distinction, the current result reassigns a role to abstract brain processes in influencing low-level non-goal-directed motor behaviours. In fact, while sensorimotor manipulation is predictably able to influence abstract thought (i.e., which number will be generated) (see [13]), the present study demonstrates how abstract information seemingly influences motor behaviour (i.e., which finger will be moved), above and beyond the awareness of one's own will. This readdresses the body-mind diatribe toward some causal reciprocity between body and mind as well as between perception and action.

This study presents some limitation. For instance I have not explored the effects of non numerical sequences (such as the exposure to letters of the alphabet) on the execution of this task. Moreover there is not information on the effects of some specific training such as musical instrument playing or on the everyday use of a computer device.

In view of these issues, future studies devoted to the investigation of this issue could explore how RMG can be affected by long-term plasticity phenomenon. For instance it could be interesting to explore RMG in Arabic or Hebrew native readers that read/write from right to left. In the context of this issue, it would be also intriguing to study the performance of bilinguals' participants (i.e., English versus Hebrew or Iranian). This would be particularly interesting in order to see whether and how the reading/writing direction suggested by the linguistic code used in the experimental session affects the performance in the RMG task. Finally, other potential future researches could explore whether RMG is affected by other forms of magnitudes such as non symbolic quantities (dots) or stimuli with different levels of luminosity.
